# Maintenance of genome integrity and active homologous recombination in embryonic stem cells

**DOI:** 10.1038/s12276-020-0481-2

**Published:** 2020-08-07

**Authors:** Eui-Hwan Choi, Seobin Yoon, Young Eun Koh, Young-Jin Seo, Keun Pil Kim

**Affiliations:** Department of Life Sciences, Chung-Ang University, Seoul, 06974 South Korea

**Keywords:** Mitosis, Cancer stem cells, Cell growth

## Abstract

Embryonic stem cells (ESCs) possess specific gene expression patterns that confer the ability to proliferate indefinitely and enable pluripotency, which allows ESCs to differentiate into diverse cell types in response to developmental signals. Compared to differentiated cells, ESCs harbor an elevated level of homologous recombination (HR)-related proteins and exhibit exceptional cell cycle control, characterized by a high proliferation rate and a prolonged S phase. HR is involved in several aspects of chromosome maintenance. For instance, HR repairs impaired chromosomes and prevents the collapse of DNA replication forks during cell proliferation. Thus, HR is essential for the maintenance of genomic integrity and prevents cellular dysregulation and lethal events. In addition, abundant HR proteins in the prolonged S phase can efficiently protect ESCs from external damages and protect against genomic instability caused by DNA breaks, facilitating rapid and accurate DNA break repair following chromosome duplication. The maintenance of genome integrity is key to preserving the functions of ESCs and reducing the risks of cancer development, cell cycle arrest, and abnormal replication. Here, we review the fundamental links between the stem cell-specific HR process and DNA damage response as well as the different strategies employed by ESCs to maintain genomic integrity.

## Embryonic stem cells: extra protection for DNA

Embryonic stem cells (ESCs), which give rise to the many specialized cells of the body, have highly effective molecular processes of DNA maintenance and repair which protect their genetic information from damage. Keun Pil Kim and colleagues at Chung-Ang University, Seoul, South Korea, review the strategies found in ESCs to maintain the integrity of their DNA as they develop and multiply. A key feature is the process of homologous recombination (HR) in which one copy of a section of DNA acts as the template allowing a damaged version of the DNA to be repaired. HR also facilitates swapping of sections of DNA when sperm and egg cells form, promoting genetic diversity. HR appears to be especially significant in maintaining ESC DNA as ESCs possess higher levels of key proteins involved in its maintenance and regulation.

## Introduction

Embryonic stem cells (ESCs), derived from inner cell mass (ICM), are capable of both self-renewal as well as pluripotency, which is the ability to differentiate into diverse cell types. In early embryonic development, the accrued mutations need to be precisely repaired to prevent chromosomal defects, infertility, or death^1^. Thus, ESCs employ specific processes that minimize the accumulation of DNA mutations to maintain their genomic integrity. In addition to prevention of the accumulation of mutations, ESCs exhibit superior capabilities to repair DNA lesions and elude the reproduction of mutations in daughter cells^2^.

It is important to understand the precise DNA repair system of ESCs, as this mechanism allows ESCs to cope with DNA lesions and helps them preserve their genomic integrity^3^. In ESCs, the constant occurrence of DNA lesions is addressed by a tightly controlled DNA damage response (DDR) system. Cells respond to damage via the immediate action of DDRs, which leads to cell cycle arrest and the expression of DNA repair genes^4,5^. Cell cycle arrest at the G1-, S-, and G2/M-phase checkpoints allows DNA repair mechanisms, such as mismatch repair (MMR), base excision repair (BER), nucleotide excision repair (NER), nonhomologous end joining (NHEJ), and homologous recombination (HR), to repair diverse types of DNA damage^6^. In the case of excessive damage, the cells activate programmed cell death, which removes ESCs from the proliferating pool^7^. However, some of these essential DDR processes, such as the G1- and intra-S-phase checkpoints, are bypassed in ESCs. In addition, high expression levels of cyclins and CDK1/2 and increased E2F1-mediated transcription of S-phase genes by hyperphosphorylation of retinoblastoma (Rb) allow ESCs to rapidly enter the S phase, resulting in a rather short G1 phase^8^. A short G1 phase helps to retain pluripotency by minimizing the induction of differentiation-stimulating signals, while the prolonged S phase permits ESCs to utilize the high-frequency HR-based error-free DNA repair mechanism operating in the S/G2 phase^9,10^.

**Fig. 1:**
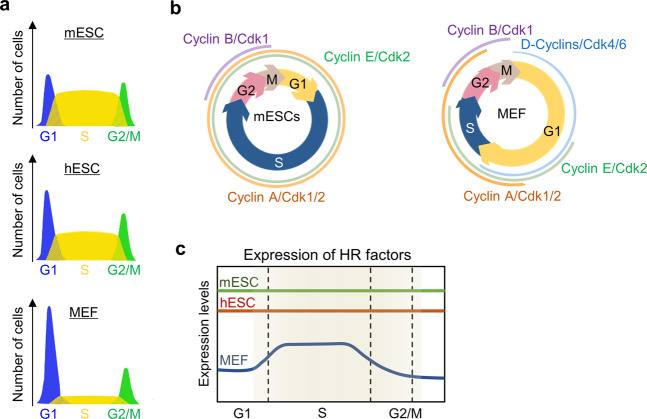
The cell cycle in ESCs and MEFs. **a** Comparison of the cell cycle patterns of embryonic stem cells (ESCs) and mouse embryonic fibroblasts (MEFs). General cell cycle profiles of ESCs (mESCs and hESCs) and MEFs are shown. **b** Differences in the expression of cell cycle components in mESCs and MEFs. In contrast to MEFs, mESCs continuously express cyclin A/E but lack the expression of cyclin D. This allows ESCs to retain retinoblastoma (Rb) hyperphosphorylation throughout the cell cycle, resulting in a rather short G1 phase. **c** Expression pattern of HR-related proteins between mES cells and MEFs. In the entire cell cycle, HR proteins are highly expressed in ESCs but not in MEFs.

ESCs preferentially utilize HR over other DNA repair pathways due to the shortened G1 and prolonged S phase compared with that of somatic cells, consequently repairing double-strand breaks (DSBs) with increased fidelity^11–15^ (Fig. 1). In particular, ESCs exhibit a relatively high expression level of HR-related proteins during the entire cell cycle^15,16^ (Fig. 1c). The elevated HR proteins, including RAD51 and RAD52, the key factors of the HR machinery throughout cell proliferation, immediately localize to DNA break sites and rapidly facilitate HR pathway activity during the S phase. It is likely that the HR process is sufficient to effectively repair aberrant DNA during the cell cycle of ESCs^15,17^. Moreover, elevated expression of HR-associated proteins can cause stable differentiation by repairing global DNA breaks induced by the unique ability of these proteins to regulate chromatin structure^15,18,19^. These specific cellular regulation systems provide genomic stability to ESCs. In this review, we summarize the DNA repair pathways that specifically function in ESCs and discuss the relationship between the stabilization of the pluripotent state and genomic integrity via the HR process.

## Regulation of the cell cycle in eukaryotes

The eukaryotic cell cycle can be classified into four phases: the Gap 1 phase (G1 phase) is the first developmental phase of the cell cycle wherein cells decide to either prepare for DNA replication or enter the resting phase (G0 phase); the synthesis phase (S phase) is the phase of DNA synthesis; the Gap 2 phase (G2 phase) occurs immediately after DNA replication and before mitosis; and the mitotic phase (M phase) involves chromosome segregation and cytokinesis, where two daughter cells are produced^20^. Each cell cycle phase is associated with a specific serine/threonine-specific kinase that controls the progression of the cell cycle by regulating substrates required in that phase^21^. These kinase proteins, also known as cyclin-dependent kinases (CDKs), are self-activated or activated by cyclins, which are key components of cell cycle regulation^22,23^. During the early G1 phase, stimulation of cells with extracellular signaling, in particular, mitogenic signaling, such as via mitogen-activated protein kinase (MAPK), by growth-promoting factors causes upregulation of the cyclin D family (D1, D2, and D3), which activates CDK 4 and 6^24,25^. In the late G1 phase, the expression levels of cyclin D and CDK 4/6 begin to decrease, while the cyclin E family (E1 and E2) of nuclear proteins and CDK2 are activated by cyclin E^24,26^. The activation of cyclin E-CDK2 leads to phosphorylation and inactivation of the Rb protein components (Rb, p107, and p130)^27^. In addition to phosphorylation of Rb, inactivation of Cdh1, one of the substrates of the anaphase-promoting complex (APC/C), induces irreversible commitment to cell division^8^. Inhibition of Cdh1 by activation of cyclin E-CDK2 at the late G1 phase inactivates the function of APC/C. This allows E2F1-mediated S-phase gene transcription and induces the progression and entry of cells into the S phase^28^. In the S phase, cyclin E may be degraded, leading to activation of the cyclin A-CDK1/2 complex, which promotes S-phase progression. During the G2/M transition, cyclin B, the key regulator of entry into mitosis, immediately translocates into the nucleus with CDK1 and phosphorylates numerous target proteins involved in the segregation of sister chromatids and cytokinesis on the mitotic spindle. Degradation of the cyclin B-CDK2 complex following mitosis denotes the start of the next G1 phase^29,30^.

## Mechanisms of DNA repair

**Fig. 2:**
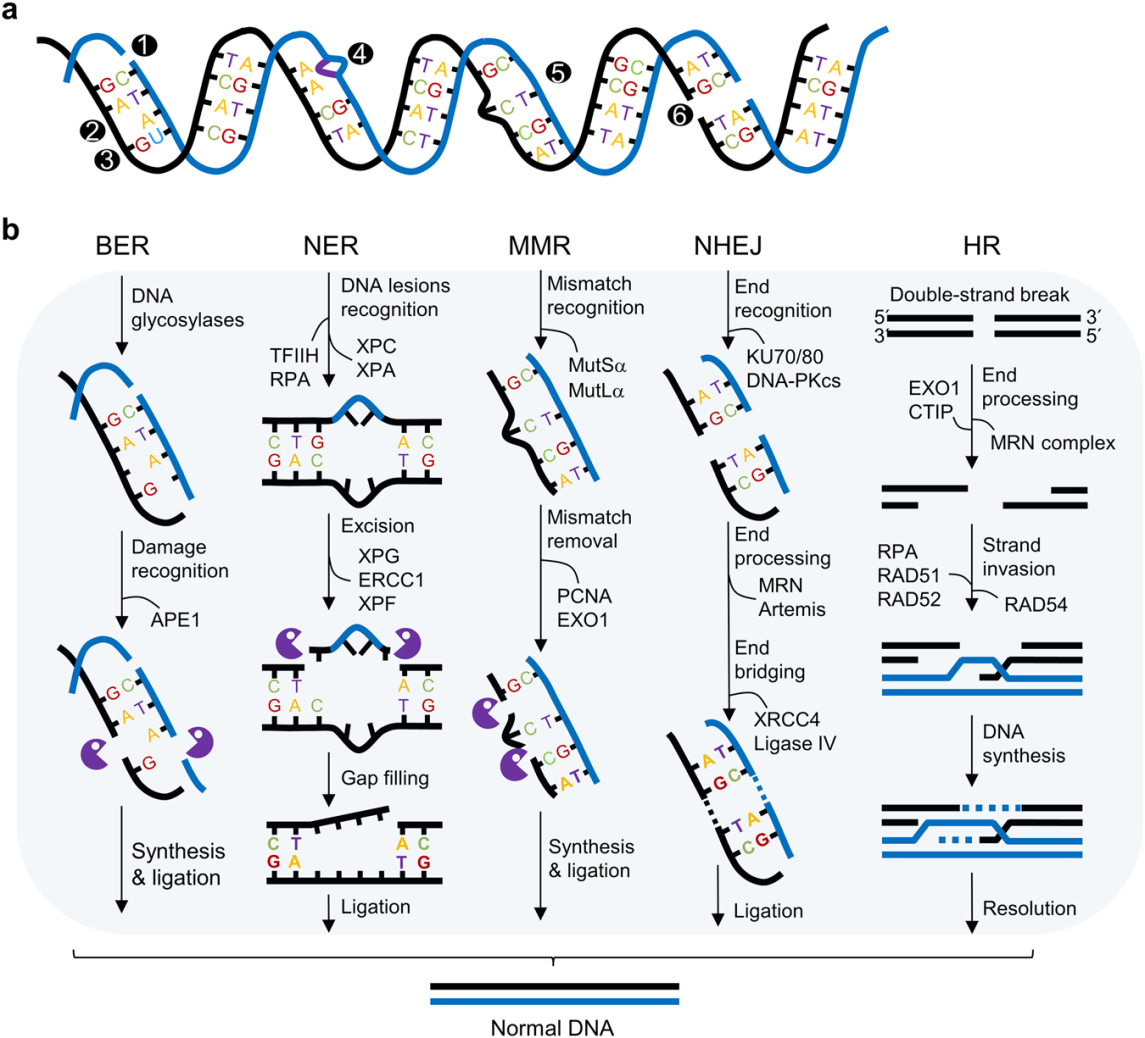
Molecular pathways of DNA repair. **a** DNA damage. (1) Single-strand breaks (SSBs), (2) apurinic/apyrimidinic (AP) sites, (3) deaminations, (4) T-T dimers, (5) DNA mismatched pairs (base to base), and (6) double-strand breaks (DSBs). **b** DNA repair processes. Various types of DNA damage can be repaired by base excision repair (BER), nucleotide excision repair (NER), mismatch repair (MMR), nonhomologous end joining (NHEJ) and homologous recombination (HR).

Cells constantly experience endogenous stress (i.e., oxidative stress during DNA replication) and exogenous stress (i.e., exposure to ionizing radiation), which can eventually lead to DNA damage (Fig. 2a). When DNA breaks occur, the DNA repair system is activated via the kinase-mediated phosphorylation of several proteins, such as ataxia telangiectasia and Rad3-related protein (ATR) and ataxia telangiectasia mutated (ATM). These two factors are activated by DNA breaks, regardless of the fact that ATR has a broad response to DNA breaks, including both DSBs and single-strand breaks (SSBs), whereas ATM primarily responds to DSBs^5–7^. Thus, both ATM and ATR are critically involved in the initiation of DNA repair under diverse DNA damage conditions in ESCs.

To maintain genomic integrity, cells cope with DNA damage by using a precise DNA repair pathway that can be generally classified into five categories: BER, NER, MMR, NHEJ, and HR (Fig. 2b). BER is initiated by DNA glycosylases that eliminate the damaged base to generate an apurinic/apyrimidinic (AP) site. This site is recognized by AP endonuclease (APE1), which initiates excision repair by DNA polymerase *β* (Pol *β*), DNA ligase III (Lig III), and X-ray repair cross-complementing 1 (XRCC1) for short-patch BER (Fig. 2). In long-patch excision repair, APE1 recognizes SSBs, which are typically repaired by DNA Polδ/ε-dependent recruitment of BER-related proteins^31^. NER is a predominant DNA repair pathway for removing bulky DNA lesions generated by UV light and environmental mutagens (Fig. 2). The damage proteins, namely, RPA, XPA, and the XPC-TFIIH complex, which bind at DNA damage sites in a random order and form a complex, recognize these lesions and initiate repair. Then, XPC is replaced by XPG in the complex. A heterodimer consisting of ERCC1 and XPF (also known as ERCC4) is recruited to the DNA lesion site, and the damaged strand is removed from both the 5´ and 3´ ends by XPG and XPF. Finally, the gaps are filled by Polδ/ε via the replication accessory factors proliferating cell nuclear antigen (PCNA) and replication factor C (RFC)^32^. MMR corrects small insertion/deletion mismatched pairs and base-base mismatches that are recognized by the MutSα heterodimer, which consists of Msh2 and Msh6 (Fig. 2). Then, MutSα recruits the MutLα heterodimer consisting of Mlh1 and Pms2 (or yeast Pms1). Subsequently, PCNA, which binds to DNA with RFC, activates MutLα to specifically incise the nascent strand. This is followed by error removal, which is generally dependent on exonuclease 1 (EXO1). DNA repair is then completed by DNA synthesis by DNA Polδ/ε, followed by ligation^33^. NHEJ is an essential DNA repair pathway that directly glues broken DNA DSB ends (Fig. 2). Therefore, the NHEJ pathway does not require a homologous sequence and is not limited to a specific phase of the cell cycle. NHEJ is initiated by the Ku heterodimer, composed of KU70 and KU80 subunits, that recognizes DSBs and binds to DSB ends^34^. The KU heterodimer directly recruits the catalytic polypeptide (known as DNA-PKcs) to the ends of the DNA to generate an active DNA-PK complex. The complex of DNA-PKcs and KU70/KU80 allows the association of Artemis with the 5´ endonuclease and processing factors, such as Pol*μ*/λ, which provide nonligatable DNA ends consistent with ligation by DNA ligase IV, XRCC4, and nonhomologous end-joining factor 1 (NHEJ1) (known as XLF)^35^. HR is an efficient DNA repair process present in all life forms. It allows high-fidelity repair of various types of DNA damage, including DNA DSBs, DNA interstrand crosslinks (ICLs), and DNA gaps. HR mainly occurs in the S/G2 phase of the cell cycle because of its dependence on a homologous template to produce paired DNA strands (Fig. 2). HR is initiated by the formation of broken DNA ends and resection from the 5´ to the 3´ end by the MRN complex consisting of MRE11, RAD50 and NBS1 (also known as Nibrin). The two short strands of ssDNA at DSBs are generated by exonucleases or endonucleases, including EXO1, and retinoblastoma binding protein 8 (RBBP8, which is also known as CTIP) to form ssDNA overhangs, which are coated by RPA to avoid DNA secondary structure formation. Then, RAD52 replaces RPA with RAD51, the recombination mediator. The resulting RAD51-ssDNA filament invades a sister chromatid to execute the homology search, and repair-associated DNA synthesis is terminated by several DSB repair networks that involve the generation of a double-Holliday junction (dHJ), which leads to crossover/noncrossover outcomes, or synthesis-dependent strand annealing^36,37^.

## Characteristics of the cell cycle of ESCs

The cell cycle represents not only a tightly organized mechanism of cell growth but also the regulation of cell fate determination. In particular, in embryonic development, cell survival and tissue homeostasis are controlled through cell cycle progression in stem cells or progenitor cells.

It has been reported that ESCs have unique cell cycle characteristics with a short G1 phase and prolonged S phase^3,20,24,38^. For example, murine embryonic stem (mES) cells, pluripotent cell lines that are derived from mouse embryos at the early developmental stage before implantation generally arise from the uterus and show a rapid cell cycle compared with that of differentiated cells^39^. In general, the estimated time for cell division in mESCs is 12 h, which is 12 h faster than the division time of somatic cells^24^ (Table 1). mESCs have a short G1 phase, which lasts for only 3 h, and a large proportion of asynchronous cells is found in the S phase (~65%) compared with the G1 phase (~15%)^40,41^. The cell cycle progression of human ESCs (hESCs) is rapid and displays a short G1 phase, as observed in mESCs. However, the regulation of the cell cycle in hESCs is slightly different. hESCs exhibit a lower proportion of cells in the S phase (~50%) than mESCs but not somatic cells (~20%) and display a longer generation time (15–16 h) than mESCs (12–13 h) but not somatic cells (23–25 h)^14,42^ (Fig. 1a; Table 1). These results show that hESCs are similar to postimplantation epiblast-derived stem cells derived from the tissue of the postimplantation embryo that produces the embryo proper^43^. Thus, many forms of pluripotent cells exist during early development.

**Table 1:** Comparison of cell cycle features (Refs. ^8,14,24,42^).

	mESCs	hESCs	MEFs
Cell division time	12 h	16 h	24 h
S-phase length	7.5 h	8 h	4.8 h
CDK1 & CDK2 activity	Very high	Very high and periodical	Periodical
RB phosphorylation status	Hyperphosphorylation	Hypo- and hyperphosphorylation	Hypo- and hyperphosphorylation
HR protein expression	+++	++	+

*mESCs* mouse embryonic stem cells, *hESCs* human embryonic stem cells, *MEFs* mouse embryonic fibroblasts

Previous studies have revealed that the expression levels of cyclins E, A, and B and the activities of CDK1 and CDK2 are elevated in ESCs compared with those in differentiated cells due to the high expression level of APC/C and its inhibitory activity against early mitotic inhibitor 1 during cell cycle progression^10,44^ (Fig. 1b). These factors allow ESCs to keep retinoblastoma hyperphosphorylated during the cell cycle and result in a rather short G1 phase^8^ (Table 1). A short G1 phase minimizes the induction of differentiation-stimulating signals and helps to retain the pluripotent state, which could be beneficial for the developing embryo^9,10^. ESCs lack a G1 checkpoint; therefore, cells damaged by exogenous stresses, such as UV radiation, are not arrested at the G1-phase checkpoint but progress to the S phase, where DNA replication occurs^45,46^. The prolonged S phase in ESCs is responsible for facilitating the accurate repair of DNA breaks by utilizing the high-frequency HR process, an error-free DSB repair mechanism functioning in the S/G2 phase^14,46^. ESCs display high levels of HR factors during the entire cell cycle. Notably, RAD51, RAD52, and RAD54 have been reported to be the key proteins of the HR process and enable ESCs to facilitate efficient replication fork progression by inhibiting replication fork collapse and repairing DNA breaks during prolonged S phases^15,17^ (Fig. 1c). Thus, ESC-specific cell cycle regulation allows cells to effectively tolerate diverse stresses, such as endogenous replication stress or exposure to exogenous DNA-damaging agents, by exploiting the DNA repair-coupled genome integrity strategy.

## Roles of HR factors in replication stress of ESCs

mESCs do not complete the replication step before moving to the S phase due to the relatively short G1 phase^5,15^. This leads to a discontinuous DNA replication process, which contributes to DSBs, which are caused by the accumulation of ssDNA gaps. Despite these weaknesses, in terms of replication, mESCs can efficiently overcome replication stress. The repair of ssDNA gaps depends on the core recombinase RAD51 and key factors associated with the HR process. The depletion of RAD51 in mESCs causes G2/M-phase arrest and replication fork collapse^3,17^. However, RPA–ssDNA nucleofilament formation is not affected by the depletion of RAD51, meaning that ssDNA gaps can be accumulated when RAD51-mediated DNA repair or gap filling is impaired^15^. Considering the abundance of key HR factors and their essential function in the S phase, it can be suggested that key HR factors abundantly expressed in mESCs rapidly localize to sites of DNA breaks or exposed ssDNA during the replication process. HR factors bind to DNA strands to prevent additional DNA break resection, which would generate unrepaired ssDNA gaps and facilitate immediate HR-regulated postreplication repair. Thus, these features strongly suggest that constitutive expression of HR factors plays a crucial role in inhibiting continuous DNA breaks by preventing the accumulation of ssDNA gaps in mESCs, which have a relatively long S phase and thus rely entirely on genomic integrity during replication.

## Regulation of the cell cycle and pluripotency

Dalton and Coverdell demonstrated that the duration spent in the G1 phase relative to the S phase can be increased by treating ESCs with roscovitine, a CDK inhibitor, which inhibits S-phase entry, disrupts the pluripotent state, and induces differentiation^47,48^. This implies that the length of the G1 phase is critical for regulating pluripotency. How might a comparatively long time spent in the S phase stabilize the pluripotent state? The essential activity of the S phase, where DNA is replicated, offers a unique chance during the life cycle of cells for epigenetic regulation and maintenance of genome stability that may be implicated in stabilizing the pluripotent state. One of the several possibilities is that the proteins directly regulating DNA replication might also stabilize pluripotency. The protein geminin is a key factor for regulating DNA replication during the S phase. This protein induces the loading of minichromosome maintenance (MCM) helicase at DNA replication origins and restrains the re-establishment of a replication origin^18,19^. Mouse embryos showing deficient expression of geminin fail to form an ICM and develop beyond the 8-cell stage^19^. In addition to its role in inhibiting replication origin re-establishment, geminin is important for maintaining the expression of the core indicators of stem cell-like capacity, i.e., Oct4, Nanog, and Sox2^49^. Aberration of the HR-mediated DNA repair system can also affect the destabilization of a pluripotent state. As ESCs undergo this process, the expression levels of RAD51 and other HR-associated proteins are reduced, as is the frequency of focus formation by HR proteins at DNA break sites^15,17,50^. In contrast, the frequency of colocalization of 53BP1 with RIF1, the suppression factors of the HR process, is increased, which suggests that the consequent recruitment of HR-associated proteins and DSB repair by the HR process are impaired^51^. Defects in HR cause a reduction in the resolution of stalled replication forks and lower the rate of formation of new replication origins, which could result in unprogrammed differentiation. A recent study has demonstrated that ectopic expression of HR-associated protein leads to stable differentiation via the repair of global DNA breaks induced by a unique ability to regulate chromatin structure, which can maintain a high rate of the open euchromatic-state genome and facilitate a repair-conducive chromatin configuration^15,52^.

## HR-mediated DNA repair pathways

**Fig. 3:**
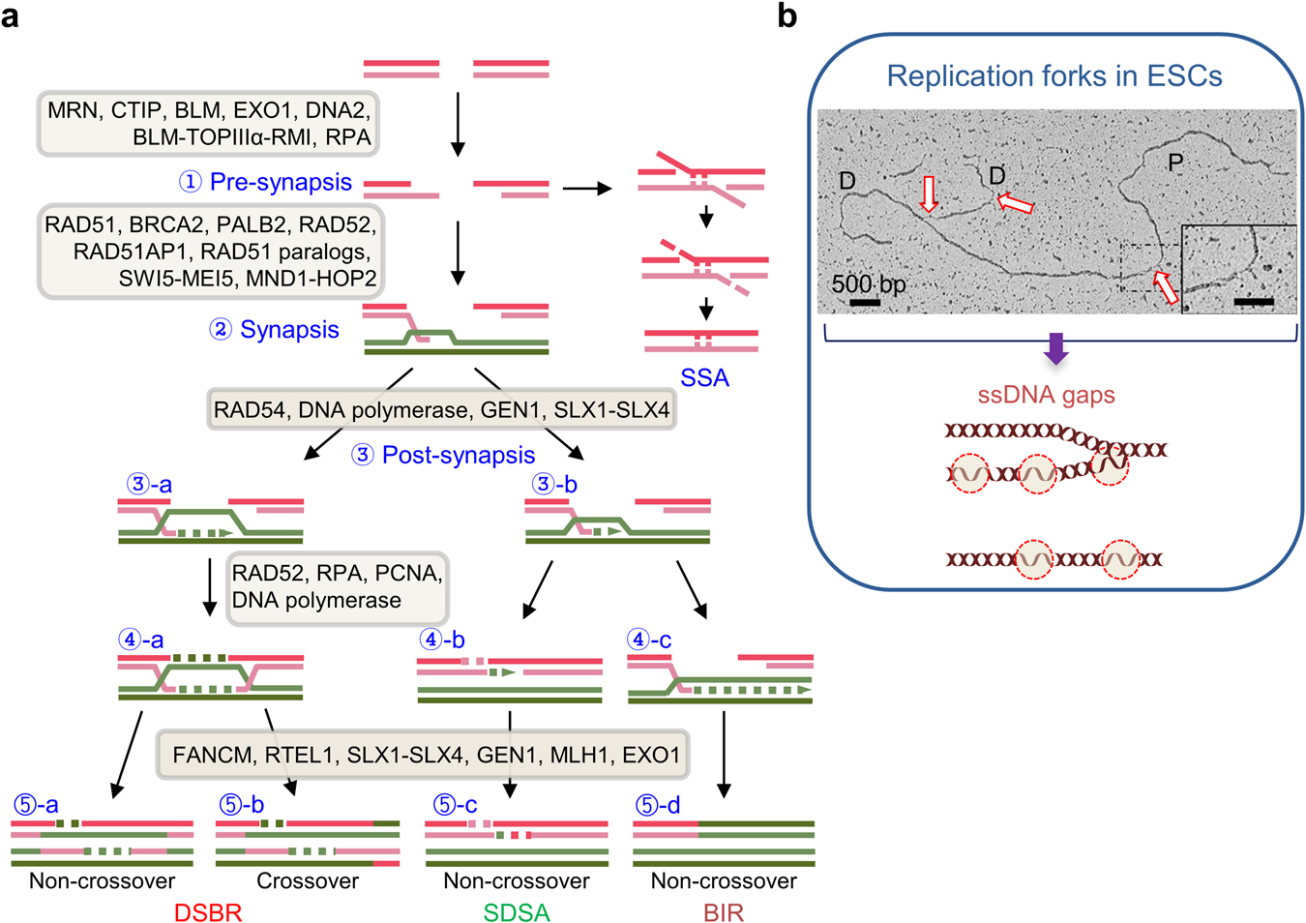
Homologous recombination promotes genome integrity in ESCs. **a** Homologous recombination (HR)-mediated DSB repair pathways. HR pathways can be generally classified into three stages: presynapsis, synapsis, and postsynapsis. In presynapsis, single-stranded tails of DSB ends are generated by nucleases (step 1). During synapsis, invasion of the DNA strand by Rad51 cofactors leads to the formation of a D-loop (step 2). At least three distinct pathways, double-strand break repair (DSBR), break-induced replication (BIR), and synthesis-dependent strand annealing (SDSA), share the D-loop intermediate. In DSBR (steps 3a-5a and 5b), DSB ends are engaged, leading to double-Holliday junction (dHJ) formation. A dHJ is a substrate for separation into either noncrossover or crossover products. In SDSA (steps 3b, 4b and 5c), the leader strand is displaced from the D-loop and reannealed with the single-stranded tail, forming noncrossover products. In BIR (steps 3b, 4c, and 5d), the D-loop is assembled into a complete replication fork and copies the entire distal arm of the chromosome. **b** Electron microscopy images of replication forks in mESCs (modified from Ahuja et al. ^3^). Arrows indicate ssDNA gaps. D, daughter DNA strand; P, parental DNA strand. The scale bar in the inset is 200 bp.

RAD51 and accessory factors play a key role in the homology search and DNA strand exchange between DSB ends and undamaged homolog templates (Table 2). HR proteins function in all three phases of the HR pathway: presynapsis, synapsis, and postsynapsis (Fig. 3a). In the presynaptic phase, RAD51 binds to ssDNA, which is generated by exonuclease action at DSB ends or from replication error. RAD51 forms a presynaptic filament in ssDNA coated with RPA. RAD52 and various axillary factors stabilize presynaptic filament assembly. This allows RAD51-mediated strand exchange. The ssDNA within the filament is then extended to stabilize the secondary structure of the joint molecules. The extension of the filament is important for precise DNA strand invasion and homology search. During synapsis, RAD51 enables the formation of a physical link between the invading DNA strand and the homologous DNA template, leading to the production of heteroduplex DNA. RAD51 double-stranded DNA (dsDNA) filaments are generated by probing both the donor and invading ssDNA strands within the filaments. Finally, RAD51 dissociates from dsDNA, and DNA is synthesized by allowing contact of DNA polymerases with the invading 3´-end during postsynapsis^36,53^. During meiosis, Dmc1, the meiosis-specific DNA strand exchange factor, promotes the formation of joint molecules (DNA strand invasion products) between homologous templates in a manner similar to that of RAD51^53^. However, it is unknown whether DMC1 and its accessory factors HOP2 and MND1 are expressed in ESCs.

**Table 2:** Homologous recombination factors.

	Human	Functions
Recombinase	RAD51	DNA-dependent ATPase activity (53)
		Homologous to the bacterial RecA (53)
		Binds to single-stranded DNA (53)
		Catalyzes the strand exchange and recognition of homology (53)
	DMC1	Meiosis-specific homology search and strand invasion (53)
		Repairs of meiotic double-strand breaks (53)
Regulators	RAD52	Promotes Rad51-dependent homologous recombination (53)
		Promotes the annealing of complementary ssDNA (18)
		Interacts with RAD51 and RPA (53)
	BRCA2	Mediates DNA strand invasion (40)
		Recombination mediator activity (53)
		Stabilizes RAD51-ssDNA filaments by blocking ATP hydrolysis (40)
	RAD51B-RAD51C RAD51D-XRCC2 RAD51D-XRCC3	Recombination mediator bound in ssDNA (51)
		Promote Rad51-dependent homologous recombination (53)
	RAD54 RAD54B	dsDNA-dependent ATPase (53)
		Involve in DNA repair and mitotic recombination (15,17)
		Stimulate of the RAD51/DMC1-regulated D-loop reaction (54)
	RAD51AP1	Promotes D-loop formation by Rad51 (53)
	SWI5-MEI5	Mediator activity (54)
	MND1-HOP2	Stabilization of RAD51/DMC1 presynaptic filaments (53)
	PCNA	Proliferating cell nuclear antigen (17)
		DNA polymerase processivity factor (17)
	PALB2	Promotes BRCA2 localization and stability in nuclear structure (40)
		Partner and localizer of BRCA2, also known as FANCN (40)
	RPA	Highly conserved eukaryotic ssDNA binding protein (19)
		Stabilization of single-stranded DNA intermediates (36)
		Activity in DNA replication, recombination and repair (36)
		Important in the process of second-end capture with RAD52 (51)
Resolution	FANCM	DNA-dependent ATPase (54)
		Helicase and strand migration activity (53)
		Resolution of meiotic recombination intermediates (54)
	BLM	Central regulator of most of the recombination events (53)
		ATP-dependent DNA helicase (53)
		Stimulates DNA Holliday junction dissolution and DNA 4-way junction branch migration (53)
	GEN1	Holliday junction resolvase (63)
		Promotes template switching (63)
	RTEL1	ATP-dependent DNA helicase (53)
		Promotes noncrossover repair by meiotic SDSA (53)
		Disassembly of D-loop recombination intermediates (53)
	SLX1-SLX4	Holliday junction resolvase (53)
		Cleaving replication fork (53)
	DNA2	ssDNA-dependent ATPase (53)
		Nuclease involved in long resection (53)
	EXO1	5′ overhang endonuclease (53)
		Long resection of DNA ends generating 3-overhangs (53)
		RNaseH activity (53)
	MLH1	Meiosis-specific dHJ resolvase in crossover (53)

HR processes can be classified into three different subpathways: DSB repair (DSBR), break-induced replication (BIR), and synthesis-dependent strand annealing (SDSA). These subpathways share the early steps, processing the DSB with a 3´-overhang tail by assembling RAD51-ssDNA on the filament. First, in the DSBR pathway, the DSBR strand proceeds to engage the second end of the DSB by either independent strand invasion or second-end capture through strand annealing, leading to the formation of a dHJ. A dHJ is a substrate either for separation into a noncrossover product by bloom syndrome protein (BLM) and topoisomerase 3A (TOP3A) or for resolution by a specific endonuclease, such as FANCM, GEN1, and SLX1-SLX4, into crossover/noncrossover products^53^ (Table 2). In mitotic DSBR, many DSBs are repaired by the SDSA pathway^54^. In SDSA, the invading strand is displaced from the D-loop and synthesized DNA with the complementary strand. Elimination of D-loops clearly requires a motor protein, such as RAD54 and BLM helicase or the SRS2 helicase (yeast)^53,55^ (Table 2). The invading strand reanneals with a single-stranded tail as in gap repair, always forming noncrossover products.

If a DSB is generated between adjacent repeated sequences, it is repaired by single-strand annealing (SSA). In the SSA process, DNA ends are stretched to form ssDNA tails, which can anneal together. The SSA pathway does not require HR proteins, as the repair pathway does not require a homology search and strand invasion, but it may require RAD52, which regulates annealing.

In the BIR pathway, the D-loop can assemble into a full replication fork and copy the entire distal arm of the chromosome, resulting in loss of heterozygosity. This process is induced more often when there is only one DNA end of the DSB, either due to the loss of the other DNA end or progression of lengthening telomeres in telomerase-lacking cells. BIR is generally dependent on the RAD51 recombinase^53,56^.

## HR promotes maintenance of genome integrity in mESCs

**Fig. 4:**
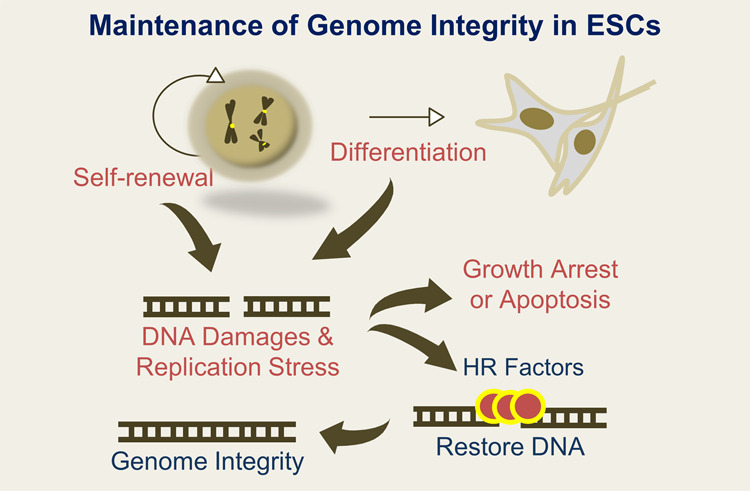
Maintenance of genome integrity in ESCs. During the self-renewal or differentiation of ESCs, abundant HR factors promote the repair of DNA damage and recovery from replication stress, thereby maintaining genome integrity.

HR is one of the major error-free DNA repair pathways. It differs from other repair mechanisms, such as MMR, BER, NER, and NHEJ. In mESCs, the expression level of HR-related factors is approximately 6-fold higher than that in somatic cells, thereby providing genome stability^15^. The abundant HR factors affect the cell cycle, which is linked to the HR mechanism, resulting in cells overcoming unstable chromatin/chromosome structures. It has been reported that genomic mutation frequency in ESCs is lower than that in somatic cells^57^. HR factors are expressed abundantly, regardless of the cell cycle phase^15,58–61^. One of the reasons is that the S-phase is longer in ESCs than in somatic cells, although the time for cell division is shorter than that in somatic cells. Strikingly, large amounts of ssDNA gaps and breaks are accumulated in replication forks in ESCs (Fig. 3b). When RAD51 is depleted, mESCs are arrested at the G2/M transition, which leads to ssDNA gaps and sensitive DNA damage, which could dramatically increase cell death. Thus, the HR pathway is required to maintain cell cycle progression and the response to stress from DNA breaks caused by long-lived ssDNAs from the prolonged S phase or DNA damage in mESCs (Fig. 4). Furthermore, the enhanced activity of HR proteins should be important for pluripotency and self-renewal in cells via maintenance of genome stability (Fig. 4). During the progression of differentiation, the HR factors RAD51, RAD52, and RAD54 tend to decrease steadily in a time-dependent manner, and cell death can be induced easily by DNA damage. Moreover, the HR factors are closely related to the cell cycle, especially the S phase. However, during mESC differentiation, the levels of HR-related factors decrease significantly. Differentiation of mESCs can cause epigenetic alteration, chromatin reorganization, and endogenous DNA breaks that could affect genome stability. In addition, ectopic expression of HR factors such as RAD51, RAD52, and RAD54 could enhance genome integrity to promote pluripotent cell differentiation^15^. This implies that the abundant HR factors facilitate cell differentiation by repairing DNA damage efficiently and quickly during chromatin remodeling.

mESCs predominantly utilize the HR-mediated repair system for DNA breaks, while somatic cells prefer to use NHEJ. When mESCs differentiation is induced, they switch their preferred process from the HR-mediated pathway to NHEJ^46^. This switch to NHEJ induces a reduction in HR-related factors, such as RAD51, that are involved in the HR-mediated process^15,46^. This leads to inefficient DNA repair, which renders mESCs incapable of protecting and mediating the replication forks, leading to a high frequency of DNA breaks via accumulation of ssDNA gaps. Notably, accumulated mutations in somatic cells reduce the possibility of conversion to induced pluripotent stem cells (iPSCs) during reprogramming, but HR factors promote active reprogramming^62,63^. Against this background, it is acceptable to hypothesize that high-frequency availability of the HR pathway facilitates stable cellular differentiation and reprogramming.

## Conclusion and future directions

ESCs exhibit unique features such as pluripotency, self-renewal, and differentiation into diverse cell types via the regulation of transcription factors. Interestingly, ESCs express higher levels of HR factors than somatic cells. This supports the maintenance of genome integrity, despite the fast cell cycle progression. Furthermore, high expression of HR factors could accelerate differentiation and decrease the increased rate of DNA breaks by repairing the breaks efficiently and rapidly. When reprogramming factors are transfected into differentiated cells with HR factors, the efficiency of iPSC generation is increased compared with that observed upon transfecting reprogramming factors alone. Therefore, HR factors could be important players in not only differentiation to somatic cells but also reprogramming to iPSCs. Further research on the HR-mediated genome integrity of ESCs could provide fundamental clues for the development of new strategies for cell therapy and provide a better understanding of the various diseases caused by the accumulation of DNA lesions in ESCs.
